# A budget impact analysis of cost to implement a whole child health focused, family-based intervention in primary care for children with elevated BMI

**DOI:** 10.1186/s43058-023-00429-z

**Published:** 2023-06-05

**Authors:** Alexandra Harris, Neil Jordan, Allison J. Carroll, Andrea K. Graham, Charlton Wilson, Fernando A. Wilson, Cady Berkel, Justin D. Smith

**Affiliations:** 1 Health Sciences Integrated PhD Program, Center for Education in Health Sciences, Northwestern University Feinberg School of Medicine, Chicago, IL USA; 2 Department of Psychiatry and Behavioral Sciences, Northwestern University Feinberg School of Medicine, Chicago, IL USA; 3 Center for Prevention Implementation Methodology, Department of Psychiatry and Behavioral Sciences, Northwestern University Feinberg School of Medicine, Chicago, IL USA; 4 Center for Behavioral Intervention Technologies, Department of Medical Social Sciences, Northwestern University Feinberg School of Medicine, Chicago, IL USA; 5 University of Arizona College of Medicine, Phoenix, AZ USA; 6 Department of Population Health Sciences, University of Utah Intermountain Healthcare, Spencer Fox Eccles School of Medicine, College of Social and Behavioral Science Department of Economics, Matheson Center for Health Care Studies, University of Utah, Salt Lake City, UT USA; 7 Population Health & Integrated Behavioral Health, College of Health Solutions, Arizona State University, Tempe, AZ USA; 8 Department of Population Health Sciences, University of Utah Intermountain Healthcare, University of Utah School of Medicine, Salt Lake City, UT USA

**Keywords:** Behavioral health, Coordinated care, Family Check-up 4 Health, Hybrid trial, Integrated care, Primary care, Pediatric obesity, Pediatric, Population health, Youth

## Abstract

**Background:**

Although the cost of implementing evidence-based interventions (EBIs) is a key determinant of adoption, lack of cost information is widespread. We previously evaluated the cost of preparing to implement Family Check-Up 4 Health (FCU4Health), an individually tailored, evidence-based parenting program that takes a whole child approach, with effects on both behavioral health and health behavior outcomes, in primary care settings. This study estimates the cost of implementation, including preparation.

**Methods:**

We assessed the cost of FCU4Health across the preparation and implementation phases spanning 32 months and 1 week (October 1, 2016–June 13, 2019) in a type 2 hybrid effectiveness-implementation study. This family-level randomized controlled trial took place in Arizona with *n* = 113 predominantly low-income, Latino families with children ages > 5.5 to < 13 years. Using electronic cost capture and time-based activity-driven methods, budget impact analysis from the perspective of a future FCU4Health adopting entity—namely, ambulatory pediatric care clinicians—was used to estimate the cost of implementation. Labor costs were based on 2021 Bureau of Labor Statistics Occupational Employment Statistics, NIH-directed salary cap levels or known salaries, plus fringe benefits at a standard rate of 30%. Non-labor costs were based on actual amounts spent from receipts and invoices.

**Results:**

The cost of FCU4Health implementation to 113 families was $268,886 ($2380 per family). Actual per family cost varied widely, as individual tailoring resulted in families receiving a range of 1–15 sessions. The estimated cost of replicating implementation for future sites ranged from $37,636-$72,372 ($333–$641 per family). Using our previously reported preparation costs (i.e., $174,489; $1544 per family), with estimated replication costs of $18,524–$21,836 ($164–$193 per family), the total cost of delivering FCU4Health was $443,375 ($3924 per family), with total estimated replication costs of $56,160–$94,208 ($497-$834 per family).

**Conclusions:**

This study provides a baseline for costs associated with implementation of an individually tailored parenting program. Results provide critical information for decision makers and a model for future economic analysis and can be used to inform optimization thresholds for implementation and, when necessary, benchmarks for program adaptation to promote scale-up.

**Trial registration:**

This trial was prospectively registered on January 6, 2017, at ClinicalTrials.gov (NCT03013309).

**Supplementary Information:**

The online version contains supplementary material available at 10.1186/s43058-023-00429-z.

Contributions to the literature

- The first study to estimate the implementation cost of Family Check-Up 4 Health (FCU4Health), a family-based pediatric obesity intervention. FCU4Health was estimated to cost an average of $482–$816 per family to implement.
- While the analysis revealed a significant investment needed to prepare for and deliver an intervention such as FCU4Health, particularly the personnel costs, results also demonstrate the relative inexpensiveness of delivering the intervention sessions.
- Few analyses have been conducted on family-based pediatric obesity interventions with this level of detail and comprehensiveness. These methods serve as a roadmap for others to facilitate cost comparisons.

## Background

Interest is growing in the potential of primary care for scaling up family-centered interventions [1–4]. These programs have demonstrated wide-ranging and long-lasting effects related to health outcomes of interest to pediatric primary care [5], including obesity and behavioral health, which have been noted by pediatricians as their biggest concerns for patients [1, 6]. In fact, the US Preventive Services Task Force recommends comprehensive, intensive, family-based behavioral interventions as an effective method for treating childhood obesity for those ages 6 years and older [7]. Despite the potential to improve child health, the uptake of family-centered interventions has lagged, perhaps due in part to uncertainty for decision makers regarding the cost of implementing such interventions in practice [1, 8, 9]. Cost is often cited as the single most important factor in the decision of whether to adopt a program [8, 9] and can vary considerably based on the target population and estimated resource use [10]. Although other family-based obesity programs may be evidence based [11], many are challenging to implement on a large scale because they are cost prohibitive for primary care and other typical service systems that might adopt them [12]. More often than not, cost data have not been collected or have been reported with limited quality or generalizability [13–15]. This stakeholder focus on costs and other financial outcomes [1, 16] is consistent with calls in the field for a greater focus on economic outcomes in implementation science [17, 18]. Moreover, economic analysis is a priority of agencies that fund implementation science and indeed was a specific focus of the Childhood Obesity Research Demonstration 2.0 funding opportunity announcement (RFA-DP-16–004) [19] that supported the current study. With respect to family-centered programs that focus on the development of parenting skills, there is some evidence to suggest cost-effectiveness or cost–benefit through a number of physical health, behavioral health, and/or socioeconomic outcomes [20–25]. However, a systematic review suggests inconsistent methods and reporting of economic analyses [26]. Our study seeks to fill these gaps by calculating the expected cost to a primary care organization to implement such programs.

### Raising Healthy Children study

In response to the recommendation by the US Preventive Services Task Force, we enhanced the evidence-based, Family Check-Up® (FCU) program [27] with obesity-related content and a whole child health approach, resulting in the Family Check-Up 4 Health (FCU4Health) [1, 28]. The FCU is an evidence-based parenting program that uses a tailored approach to address child behavioral health problems [27]. The program includes a comprehensive assessment to guide services, motivational approaches to engage families, and connections with community resources [29], including an accompanying parent training curriculum [30]. Berkel et al. [1] and Smith et al. [28] provide descriptions of the FCU4Health program and the process and stakeholders involved in adapting the FCU for obesity prevention and management in coordination with pediatric primary care.

Guidance from our community partners demonstrated that a family-centered program that explicitly focused on both physical and behavioral health outcomes would be more appropriate for the primary care context than a program focused solely on behavioral health outcomes: Our findings indicate pediatric practices are more interested in training and supporting a single evidence-based intervention that has transdiagnostic effects rather than having to support multiple programs for specific presenting issues (e.g., a program for behavioral health and another program for obesity/health behaviors) [1, 31, 32]. Consequently, adaptation of the original FCU was undertaken to (1) frame the program as health promotion (rather than risk reduction) and take a whole child approach to care, (2) assess obesity-related health behaviors, including nutrition, physical activity, sleep, and family health behaviors/routines, (3) develop family goals for health and health behaviors, and (4) coordinate with health-related resources in the community [28]. Moreover, stakeholders (e.g., administrative and clinical staff from pediatric primary care clinic; representatives from Medicaid, the Department of Health and Human Services, and health plans; researchers with expertise in pediatric obesity, health disparities, and family engagement) were engaged in the adaptation and implementation of the resulting FCU4Health in primary care settings [1, 32].

The Raising Healthy Children project is a type 2 hybrid effectiveness-implementation study [33] using a family-level randomized trial design (FCU4Health or services-as-usual) to test the implementation and health impacts of the FCU4Health intervention for children with elevated body mass index (BMI) for age and gender [34]. Eligible families were randomized at a 7:5 ratio to FCU4Health (*n* = 141) or primary care-as-usual (*n* = 99) and were stratified by child age, gender, and race/ethnicity. The primary effectiveness outcomes were child obesogenic health behaviors and family health routines; secondary outcomes were child behavioral health and parenting behaviors. Results demonstrated significant effects on child health behaviors (diet, eating behaviors, physical activity), child behavioral health (internalizing and externalizing behaviors), and child self-regulation, as well as family health routines (mealtimes, screen time, sleep) and parenting (limit-setting, proactive parenting, and parental warmth) [35–37].

Implementation occurred in partnership with primary care agencies in Maricopa County, Arizona. The Raising Healthy Children project began as a comparative (non-randomized) implementation trial of two delivery models for family-based parenting intervention and pediatric primary care: referral-based and integrated/co-located care. Although all clinics in both arms enrolled families and provided FCU4Health, it became clear after 6 months of implementation that the referral model was generating far greater enrollment, appeared to be more feasible, and addressed several of the barriers experienced by the integrated/co-located care sites (e.g., staff availability) that limited prospects for sustainment. At that time, all participating (*n* = 3) clinics moved to the referral model with support from our funder under the cooperative agreement (U18) mechanism and we expanded the number of primary care partner agencies to *n* = 7 total who would refer families.

Several strategies were used to support implementation of the referral model, including (1) interventionist training, consultation, and certification processes; (2) identification during patient visits and case finding using the electronic health record; (3) a workflow analysis of a tailored implementation plan for each clinic [38]; and (4) flexible delivery approaches (while maintaining fidelity to the intervention), with a dual delivery strategy of local, clinic-based visits and home visitation Although FCU4Health sessions were primarily delivered in-home, coordinators and families could also meet at community-based locations (e.g., public library, local foundation that provided an office space for meeting with families) rather than in clinic space. Additional information regarding implementation strategies and the implementation protocol are described elsewhere [35, 36, 39].

The economic aim of this trial is guided by the Exploration, Preparation, Implementation, and Sustainment (EPIS) model [40], particularly the latter three phases, as details about the cost incurred to an organization prior to project team involvement are unknown and likely less relevant to decisions about adoption. In a prior study, our team conducted a budget impact analysis to calculate the costs associated with FCU4Health during the implementation Preparation phase (i.e., those costs incurred in the preparatory work of implementing prior to offering the program to eligible families) [41]. Breaking out the costs in this way is important given the evidence that approximately half of all entities that begin the process of adopting a new intervention fail to implement with the intended recipients [42], meaning that preparation costs are sunk and might never be recouped under some reimbursement and financing models. Further, preparation costs and implementation costs do not always occur in the same budget year, so separating them out provides more accurate information for adopters for planning purposes. We found that the total costs of preparing to implement the FCU4Health in three clinics within the context of the Raising Health Children randomized trial was $174,489 (inflation adjusted to 2021 US dollars [41] and automated coding cost development has been removed from prior published results). A significant proportion of these costs was attributed to personnel time spent developing and tailoring clinical materials and training the FCU4Health coordinators. Given the bulk of costs were associated with the initial development of the intervention delivery and monitoring materials, we estimated that the costs to prepare to implement the FCU4Health in subsequent pediatric primary care systems would range from $18,524 to $21,836 ($164–$193 per family) (also inflation adjusted value to 2021 dollars).

### The present study

This study estimates the budget impact of the Implementation phase of FCU4Health in the Raising Health Children trial from the perspective of the pediatric primary care system. We first provide results of the actual costs of implementing and delivering FCU4Health in this trial and then estimate the range of costs needed to replicate this effort, to distinguish between costs only associated with this initial implementation versus those that are likely to be incurred by future adopting pediatric primary care systems. Finally, we sum implementation preparation costs from our prior analysis [41] and costs during the FCU4Health Implementation phase to estimate the total cost in this setting.

## Methods

### Participants

Those involved in implementing the FCU4Health included the leadership and clinicians of the primary care sites, members of the community advisory board (CAB), and the university-based research team. The research team included program developers, expert consultants/supervisors, information technology and data support staff, the interviewers who conducted the assessments, and independently licensed behavior health coordinators who delivered FCU4Health. Because this study was the first trial of FCU4Health, the CAB assisted in adapting the program to this intervention’s focus on obesity-related health behaviors and helping the research team integrate with the local primary care system prior to implementation [1]. During implementation, the CAB advised on ongoing modifications to the implementation strategy. Finally, the implementation sites were engaged to test the feasibility of collaborating with local clinics that serve significant proportions of low-income and Latino families, who are disproportionately burdened by obesity.

### Time horizon

This study reports on the costs over 32 months and one week: The implementation preparation phase of FCU4Health occurred from October 1, 2016, through April 14, 2017, when enrollment of families into the trial began; the Implementation phase of FCU4Health occurred from April 14, 2017, to June 13, 2019.

### Analytic framework

The perspective for the budget impact analysis was that of a future FCU4Health adopting entity, to provide realistic cost information to inform budgeting by adopting organizations and comparisons with the cost of alternative approaches and programs. Our analytic approach and reporting framework—namely, determining both labor and non-labor costs using an ingredients-based cost calculator approach—followed the guidelines set forth in the Principles of Good Practice for Budget Impact Analysis [43]. In alignment with our study of implementation preparation costs [41], the cost calculator used in this study was developed using Microsoft Excel and specific to the implementation of FCU4Health. Cost breakdowns are provided for each of the strategies and major activities that supported implementation. Costs captured in the analysis include both direct budgetary expenditures and the opportunity cost associated with uncompensated activities carried out as part of implementation.

### Input data

#### Costs of implementing FCU4Health

Input data included labor and non-labor costs associated with implementing FCU4Health; these data are reported in Tables 1 and 2. Labor costs (including both fixed and variable labor costs) included time spent on implementation activities carried out by the research team, CAB members, and implementation site members. To calculate a per-hour salary amount for each individual, fixed labor costs were derived by cross-walking their position title and degree with 2021 Bureau of Labor Statistics (BLS) Occupational Employment Statistics, National Institutes of Health-directed salary cap levels for academic employees at or above the cap value, or known annual salaries, plus fringe benefits at a standard rate of 30% [44]. These values were applied to each individual’s time spent engaged in each implementation activity (Table 2) and then aggregated across these activities to derive estimates of the time spent and cost associated with completing each activity. Variable labor costs as a function of families served were calculated by summing costs associated with direct intervention delivery, including travel costs, facilitator/family meeting time, and assessment delivery (Table 2). Though 113 families received the intervention, only 92 families had complete data to estimate variable labor costs. As a result, we imputed and applied mean value estimates for 21 families who had missing or incomplete data, based on the actual values for 92 families. Non-labor costs included equipment, software, and supplies and were based on actual amounts spent, which were tracked with receipts and invoices. Because our budget impact analysis uses a short time horizon, overhead costs are fixed in the short-term. As such, it is customary to exclude them from the analysis [45].

**Table 1:** Budget impact analysis labor and non-labor input parameters

Variables	Input parameter	Reference
*Average salaries*		
Implementation support/research team	$122,676	BLS 2021 and actual salaries
Community advisory board (CAB)	$156,572	BLS 2021 and actual salaries
*Hardware*		Purchasing order receipts
iPads	$12,480	
Microphones	$312	
Stadiometers	$352	
*Software*		
Portal	$1244	
Software license	$ 3600	
VZW data plan (mobile services)	$9461	
Verbalink transcription service	$1328	
*Supplies*		
Manuals	$247	
Printing	$581	
iPad cases	$315	
Extension cables	$56	
Tripod mount	$31	
Tripods	$48	
EDP Books	$393	
Pack n roll portable carrier (for all assessment equipment)	$115	
*Additional non-labor costs*		
CAB meeting costs (parking/food)	$2736	
**Total**	$33,299

**Table 2:** Labor hours and costs of implementation

Labor activities	*N*^a^	*N*^a^	Imp prep hours	Imp hours	Total hours	Total imp prep hours (%)	Total imp hours (%)	Hours by support team (%)	Hours by support team (%)	Total imp prep cost	Total imp cost		Total cost	Total imp cost (%)	Costs by support team^b^ (%)	Costs by support team^c^ (%)	
***Direct intervention delivery (variable costs)***																	
Facilitator/family meeting time (FACL hours)	**-**	**-**	-	397	397	-	7%	-	100%	-		$10,865	$10,865	5%	-		100%
Assessment (waves 1–4)	**-**	**-**	-	1270	1270	-	22%	-	100%	-		$28,184	$28,184	12%	-		100%
Mileage reimbursement (families/facilitators)	**-**	**-**	-	-	-	-	**-**	-	**-**	-		$10,138	$10,138	5%	-		100%
Other travel costs by implementers (time traveled to sessions)	**-**	**-**	-	-	-	-	**-**	-	**-**	-		$2248	$2248	1%	-		100%
***Intervention delivery infrastructure and planning***																	
Developing or tailoring intervention materials/assessment tech	9	9	662	1442	2104	22%	25%	100%	100%	$24,260		$39,776	$64,036	17%	16%		100%
Meetings regarding clinical activities	12	8	141	94	235	5%	2%	92%	100%	$6701		$4408	$11,109	2%	4%		100%
Consultation preparation	-	5	-	203	203	-	3%	-	100%	-		$13,523	$13,523	6%	-		100%
Coach consultation	-	1	-	64	64	-	1%	-	100%	-		$2473	$2473	1%	-		100%
***Training and supervision***																	
Tailoring training materials	10	6	391	83	474	13%	1%	90%	100%	$17,292		$3289	$20,581	1%	11%		100%
Meetings regarding training activities	11	8	114	130	244	4%	2%	91%	99%	$6051		$6222	$12,273	3%	4%		99%
Consultation/supervision	1	12	2	225	227	< 0.1%	4%	100%	100%	$125		$11,260	$11,385	5%	< 0.1%		100%
Participating in training	23	5	520	122	672	17%	2%	48%	100%	$22,586		$6502	$29,088	3%	15%		100%
***Materials***																	
Reviewing new or revised materials	11	7	107	125	232	4%	2%	100%	100%	$4784		$4982	$9766	2%	3%		100%
***Administrative delivery support***																	
Meetings about delivery plan	49	12	281	417	698	9%	7%	18%	96%	$17,756		$20,375	$38,131	9%	12%		95%
Developing infrastructure to support delivery	7	3	81	78	159	3%	1%	100%	100%	$4164		$3222	$7386	1%	3%		100%
***Administrative***																	
Email communication	15	11	328	500	828	11%	9%	73%	99%	$16,153		$22,870	$39,023	10%	11%		99%
Hiring clinical staff	6	3	44	18	62	1%	< 0.5%	100%	100%	$3059		$769	$3828	< 0.3%	2%		100%
***Practice engagement/community advisory board***																	
Meeting time	-	98	-	694	-	-	12%	-	49%	-		$44,483	$44,483	19%	-		49%
**Total**	-	-	2671	5860	8531		100%	-	-	$122,931		$235,587	$358,518	100%	-		-

*Imp Prep*, implementation preparation phase activities; *Imp*, implementation phase activities. Imp Prep phase costs are those reported in Jordan et al. 2019, *Prev Sci* but have been inflation adjusted to 2021dollars to match Imp phase costs

^a^Individuals can be counted in multiple categories

^b^13% of hours and 11% of costs were accrued by the clinic staff, and 1% of hours and 2% of costs were accrued by the community advisory board

^c^4% of hours and 5% of costs were accrued by the clinic staff, and 2% of hours and 5% of costs were accrued by the community advisory board

#### Estimated costs needed to replicate implementation preparation

Because this was the first implementation trial of FCU4Health, some activities required more time than would be anticipated for future adopters. Consistent with our study of implementation preparation costs [41], we estimated the proportion of time needed to replicate the implementation activities with subsequent agencies (Table 3). The estimates for conducting this replication analysis were based on feedback from the members of our CAB, staff from the delivery sites, and the multiple principal investigators of the study (CB and JDS) meeting and reaching consensus on these estimated values and ranges for each type of cost included in the budget impact analysis. For example, some activities would not need to be included in future efforts to the same extent as they were in this trial (e.g., developing materials, assessment programming) while others might require more time (e.g., training and supervision depending on the skill level of the clinicians tasked with delivery of the FCU4Health). From these values, we determined the total labor hours and costs associated with replication.

**Table 3:** Assumptions of estimates for replication input parameters for sensitivity testing

	**Estimate**	**Range**
**Labor costs**		
Developing or tailoring clinical materials	25%	15–30%
Meetings regarding clinical activities	50%	40–60%
Supervising delivery to pilot families	100%	100–200%
Developing or tailoring training materials	25%	20–35%
Meetings regarding training activities	25%	0–25%
Participating in training	90%	65–90%
Reviewing new or revised materials	25%	10–25%
Meetings to develop a delivery plan	35%	15–45%
Developing infrastructure to support delivery	30%	15–55%
Communicating by email	50%	40–60%
Hiring clinical staff	100%	75–105%
**Non-labor costs**	**Needed for replication?**	
iPads (*n* = 20)	Required	
Microphones (*n* = 20)	Optional	
Scales (*n* = 10)	Required	
Stadiometers (*n* = 10)	Required	
Portal	Required	
Software license	Optional	
Manuals	Required	
Printing	Required	
iPad cases (*n* = 23)	Optional	
Extension cables (*n* = 20)	Optional	
File folders	Required	
Tripod mount (*n* = 2)	Optional	

### Data collection

Over the duration of FCU4Health delivery, members of the university-based research and implementation site teams completed an electronically administered cost capture template developed for this study to document the number of hours they spent engaging in clinical, non-research activities (defined in Additional file 1, which also includes definitions of Preparation and Implementation phase activities). Time spent on direct delivery of FCU4Health was also monitored at the clinic sites by administering an electronic session tracking checklist. The research team used meeting attendance records to document the number of hours members of the CAB and individuals at the clinic sites spent participating in meetings and trainings related to FCU4Health implementation. Respondents were instructed to only report time spent on activities that directly supported implementation and not to report time spent on research-related activities. Research-related activities were not included as options in the cost capture template.

### Analyses

We used descriptive analyses to estimate the total number of labor hours and both labor and non-labor costs of implementation, based on unique labor-driven implementation activity categories outlined in Table 2. These labor activities (including fixed and variable) were organized under the following categories: direct intervention delivery, intervention delivery infrastructure and planning, training and supervision, materials, administrative delivery support, administrative activities, and practice/CAB engagement. Additional information about each implementation activity can be found in Additional file 1. Additionally, exploratory descriptive analyses were conducted to estimate the cost of future efforts to replicate FCU4Health implementation. Given that these values are estimates and were not prospectively measured, we tested the sensitivity of the replication estimates by varying the values across a reasonable range for each cost category. Both the estimates and the associated ranges for labor costs were based on the implementation plan to be used in future FCU4Health adoption and our experience in this study. Finally, to calculate total FCU4Health implementation costs, we added costs from the Preparation phase reported by Jordan et al. [41] to the implementation costs calculated in this analysis.

## Results

### Costs of implementing FCU4Health

#### Non-labor costs

Non-labor costs are presented in Table 1 and summed to $33,299 ($295 per family). Most non-labor implementation costs were for purchasing iPads (used for the administration of the family health routines assessment) ($12,480; 38% of total non-labor costs), followed by the data plan for mobile services for iPad to send the acquired responses immediately to the university’s research data server to prevent data loss and to ensure protection of human subjects data ($9461; 28%).

#### Labor hours and costs

Total labor hours and costs associated with the implementation are presented in Table 2. Total labor hours summed to 5860, and total labor costs summed to $235,587, or $2085 per family. Ninety-nine individuals participated in at least one implementation activity: 25 (25%) were members of the university-based research team, 26 (26%) were members of the CAB, and 48 (49%) were staff at clinic sites. Nearly all labor hours were accrued by the research team (5486 h, 94%), followed by clinic staff (235 h, 4%) and the CAB (139 h, 2%). Labor costs were also primarily attributable to the research team ($211,434, 90%), followed by CAB members ($11,660, 5%) and clinic staff ($12,494, 5%).

The most labor-intensive implementation activity was tailoring of intervention materials and assessment technology (1442 h, 26%), followed by administration of the family health assessments (1270 h: 22%). In terms of implementation labor costs, meeting time ($44,483, 19%) and tailoring of intervention materials and assessment technology ($39,776, 17%) were the two costliest activities.

### Estimated replication costs for implementation

We estimated the proportion of effort that would be required to replicate FCU4Health implementation, including both labor and non-labor costs. Table 3 indicates which equipment or supplies are required, optional, or could be substituted for an alternative, often less expensive version during future implementation. The estimated replication costs ranged from $37,636 to $72,372 ($333–$641 per family).

**Fig. 1:**
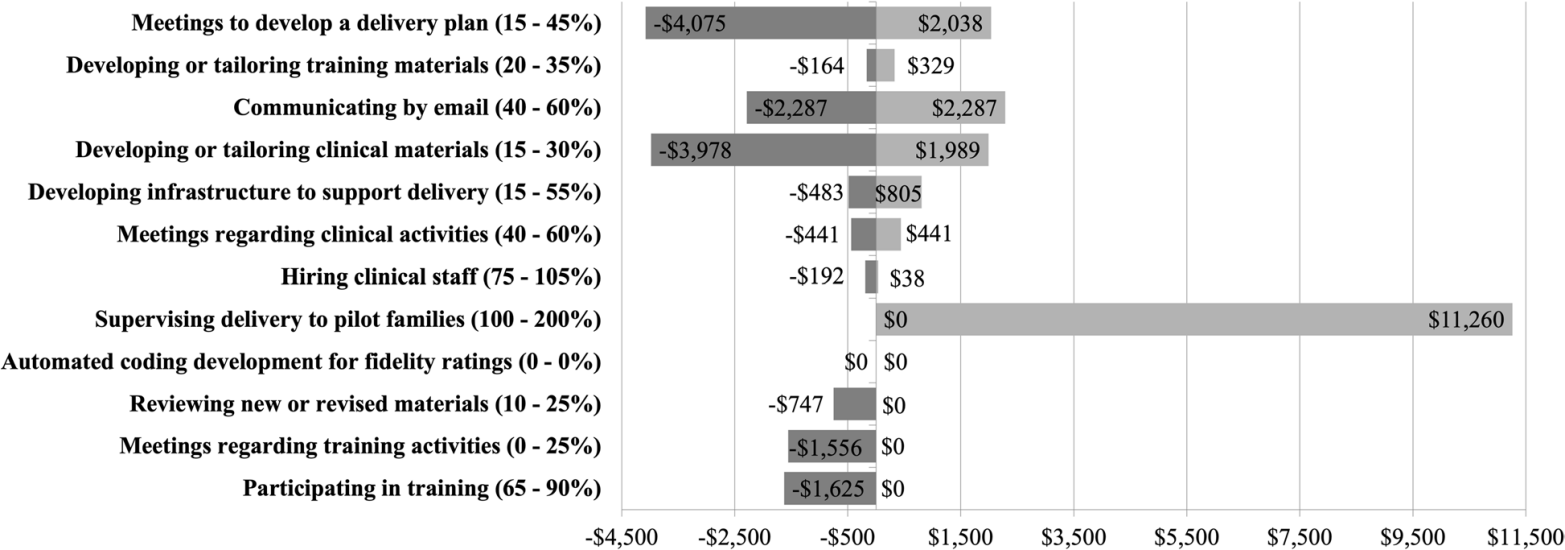
Sensitivity testing around replication estimates for each implementation labor activity

Figure 1 provides a visual representation of the results of sensitivity testing for each labor activity replication estimate and range. As shown in the figure, the activity with the greatest variation was participation in meetings to establish a delivery plan, as the replication estimates range from increasing the observed costs by $2038 to reducing them by $4075. This range is due to the wide variation in potential implementation contexts of pediatric primary care. For example, some contexts may require more frequent meetings with a larger group of stakeholders, which may increase costs. Supervising delivery of the intervention is the activity most likely to increase implementation costs ($11,260), which would vary by the skill level of the clinicians and the frequency of the supervisory model used. Reducing the time spent participating in meetings to establish a delivery plan ($4075) and developing/tailoring clinical materials ($3978) would lead to the largest reductions in implementation costs. Compared to this trial implementation, replication estimates indicate that future adopters could reduce labor costs by more than $15,000 across all labor activities or increase labor costs by more than $19,000.

### Total implementation preparation and implementation costs

To provide a comprehensive estimate of the total costs associated with implementation of the FCU4Health program, we summed the estimated costs from the Preparation phase, previously published by Jordan et al. [41], and the estimated costs from the Implementation phase presented above. Combining the preparation costs and the implementation costs, the total cost of implementing FCU4Health was $443,375, with $174,489 (39% of total costs; $1544 per family) attributed to Preparation phase costs and $268,886 (61% of total cost; $2380 per family) attributed to Implementation phase costs. Across both phases, combined total labor hours summed to 8871, and total labor costs summed to $358,518, or $3173 per family. Nearly all of the labor hours (8076 h, 91%) and labor costs ($324,717, 89%) were accrued by the research team [41]. Summing across both phases, the total estimated costs for replication are $56,160-$94,208, an average of $497-$834 per family to implement.

## Discussion

With evidence that FCU4Health is both effective and appropriate for use in primary care, there has been increasing interest in its adoption in primary care settings. It is noteworthy that most of the components of the program fit within the comprehensive set of services delivered by Federally Qualified Health Centers (https://www.hrsa.gov/opa/eligibility-and-registration/health-centers/fqhc/index.html) and most services may be reimbursable to Medicaid providers, and at no cost to recipients, through the Early Periodic Screening, Detection, and Treatment benefit or through payment innovation and demonstration models under provisions of the Affordable Care Act [46], which often results in widespread coverage by other insurers [47, 48]. Given that cost is often cited as the single most important factor in the decision of whether or not to adopt a program [8, 9], we conducted a prospective budget impact analysis of the Implementation phase costs of FCU4Health in primary care for 113 families who engaged with the program.

Our results indicated that the cost associated with the Implementation phase of FCU4Health was $268,886, or $2380 per family. Meeting time, tailoring intervention materials, and assessment technology were the largest drivers of labor costs. Non-labor costs ($33,299) were driven by the cost of iPads and mobile data plans for secure data collection. This is important from an implementation equity perspective, given that many high resourced primary care settings will already have iPads and Wi-Fi that can be used for program delivery, while these resources are likely to be limited in under resourced settings. The total cost of implementing FCU4Health (including Preparation costs from Jordan et al. and Implementation costs reported in this study) was $443,375 ($3924 per family).

Our cost estimates are comparable to those in other published cost estimates (converted to 2021 dollars using the BLS CPI Inflation Calculator to facilitate comparison) [49]. For example, an efficacy study of the original Family Check-Up (FCU) program, not adapted for health behaviors or obesity management, with children ages 2–5 reported an average cost of $4714 per family [10]. Few childhood obesity programs are comparable to FCU4Health. We discuss two programs that conducted rigorous cost assessments that provide a range. However, the cost estimates of these programs are driven in large part by the differing number of sessions. Implementation costs for High Five for Kids, a parent-focused obesity program for children aged 2 to 7 with overweight or obesity that consists of four in-clinic visits and two phone calls, were $66,562 or $302 per child [50]. This intervention differed from FCU4Health implementation in that it was clinic-based as opposed to home visiting and had a fixed number of intervention sessions for each family. Second, a parent-only treatment (for parents of children with overweight or obesity) that consisted of over 20 visits in a 6-month period and included diet and physical activity recommendations, parenting skills, and behavioral therapy components had average costs per parent–child dyad of $3248 [51]. This intervention had nearly three times the number of average sessions per family compared to FCU4Health in our study. Additional important information about the implementation costs of these studies is not reported to make a more detailed comparison of reasons for differences.

Variation in the cost of implementation could be attributable to many factors, including the total number of sites or participants involved (e.g., economy of scale) and the degree of implementation support needed because of the complexity of the intervention itself, the population involved in the intervention, and the skill level of the delivery agents. Kuklinski et al. [10], for example, found decreasing delivery costs over 4 years of delivery of the FCU, due in large part to families participating in fewer interventions sessions in later years, but also likely due to less need for implementation support during what might be considered a sustainment phase of delivery.

The variable costs of delivering FCU4Health accounted for only 19% ($51,435) of the total costs that occurred during the Implementation phase of the project. This is important to underscore for two reasons. First, it provides compelling evidence for the need to conduct comprehensive cost analyses of implementation strategies and not just the direct delivery of the intervention. Second, it points to the potential economy of scale of FCU4Health given that the time required for many of the implementation support activities would be relatively constant regardless of the total patients in the program. Future analyses may seek to model the ideal number of families to maximize economy of scale. For organizations using a fee-for-service model, maximizing direct service hours is critical, as these are the only hours that are reimbursable by health insurance. The goal of being efficient and maximizing potential benefit to patients remains a priority for accountable care and other bundled payment models as well. In a similar vein, future research ought to consider designs capable of determining optimal dose and type of implementation support required for high-fidelity, effective delivery from a cost perspective.

This study does not include a focus on the cost relative to outcomes or the return on investment for clinics, which may also contribute to scale-up decisions. The economic impact of childhood obesity is substantial and long lasting for families, the healthcare system, and society at large. Elevated BMI among children is associated with over $14 billion annually for prescription drug, emergency department, and outpatient visits [52]. The incremental lifetime medical cost, starting at age 10, for a child with obesity is $19,000 per child higher than for a child without obesity [7]. Given these statistics, the cost of implementing programs like the FCU4Health is likely justified but needs to be examined. Moreover, the original FCU program is estimated to have approximately a $198 benefit-to-cost ratio [12], perhaps indicating opportunities for the adapted FCU4Health program to yield a positive return on investment when scaled up. This impact will be assessed in a future study.

A challenge preventing widespread adoption of family-based pediatric obesity programs like FCU4Health, regardless of cost-effectiveness, is the lack of reimbursement from insurance companies [53]. These programs do not necessarily align seamlessly with payment structures, in that billing codes that could be used for these types of interventions often do not exist. However, in the case of this FCU4Health intervention, study sites used appropriate billing codes (Current Procedural Terminology [CPT] codes) for covered services to submit claims to insurance companies for reimbursement. Future research could more closely examine how and to what degree different aspects of the FCU4Health program could be reimbursed under different reimbursement models, including fee-for-service and value-based care, to inform evidence-based financing strategies that are sustainable [53, 54].

### Limitations and future directions

First, labor costs were calculated primarily based on 2021 BLS Occupational Employment Statistic salary estimates with a fringe rate of 30%. Because we aligned study roles to BLS job codes, these estimates may deviate from true salaries. This limitation is unique to this implementation of FCU4Health; future adopters may have more concrete salary information with which to make more accurate projections. Second, labor cost estimates for CAB members who did not have a second role on the project were based solely on meeting attendance, which could have underestimated their contributions if they engaged in other activities supporting the project. Third, while time spent on specific implementation activities was recorded via weekly surveys by those involved, there could be some missing data despite efforts to follow-up and prompt respondents when no survey was submitted for a particular week. Thus, it is possible that these estimation methods may not fully capture effort spent on the project. Fourth, when calculating variable costs related to direct intervention delivery (e.g., travel costs, mileage reimbursement, facilitator/family meeting time), we imputed and applied mean value estimates for 21 families who had missing zip codes, based averages from the actual values for the 92 families with zip codes. These estimated costs comprised about 7% of the total implementation labor costs; therefore, we do not believe this imputation had a substantial effect on the overall cost estimate. Fifth, given this is the first implementation trial of FCU4Health, these results are best viewed as projections when applied to plan future efforts. Similar implementation costing of the program in subsequent efforts will be needed to estimate actual costs to future adopters.

We acknowledge that not all stakeholders (e.g., members of our CAB) were consulted during the interpretation of these cost data; only co-author CW, who at the time of the project was Chief Medical Officer of a local health plan and a member of the CAB. Nonetheless, all stakeholders on the CAB and at implementing sites were consulted regularly, both when preparing and implementing the intervention, and were also involved in the development of the cost capture survey to ensure that we were collecting data on activities salient to the project. The implementation model evaluated for the majority of participants in this study (referral from primary care to an external FCU4Health service) is but one of a few ways that the program could be embedded in pediatric primary care. Budget impact analyses with a larger number and more variable sites would help to distinguish across the behavioral health integration continuum [55], though it is expected that the majority of the cost estimates here would be similar. Finally, this study only reports two phases of EPIS [40] as these corresponded to the funded study period. This did not allow for cost data collection of the Exploration phase (as this work was already completed) and the grant ended prior to Sustainment of FCU4Health. Future studies should collect rigorous cost data across all phases of EPIS.

## Conclusions

This budget impact analysis estimated that total costs of both preparation and implementation activities were $443,375, with $174,489 (39%) attributed to preparation costs and $268,886 (61%) attributed to implementation costs—an average of $3924 per family. FCU4Health scale-up costs will likely be lower because some costs will be significantly reduced or eliminated in future implementations. The estimated cost for replication of FCU4Health implementation is approximately $497 to $834 per family based on sensitivity analysis of estimated cost parameters. The analysis presented here provides decision-makers and future adopters with comprehensive cost information related to implementing an effective family-based pediatric obesity management program in coordination with primary care. Additional economic analyses focused on return on investment, cost effectiveness, and other budget perspectives, such as the cost to families, should be considered.

## Supplementary Information

**Additional file 1.** Description of implementation activities.

## Data Availability

Data and materials are available upon request to the corresponding author.

## Abbreviations

BLS: Bureau of Labor Statistics
BMI: Body mass index
CAB: Community advisory board
EBI: Evidence-based intervention
FCU: Family Check-Up
FCU4Health: Family Check-Up 4 Health program
FQHC: Federally Qualified Health Center
